# 'Toothless’—the absence of political priority for oral health: a case study of Ireland 1994–2021

**DOI:** 10.1186/s12903-022-02125-1

**Published:** 2022-03-27

**Authors:** Úna McAuliffe, Helen Whelton, Máiréad Harding, Sara Burke

**Affiliations:** 1 School of Public Health, University College Cork, 4th Floor, Western Gateway Building, Western Rd., Cork, T12K8AF Ireland; 2 Oral Health Services Research Centre, University Dental School and Hospital, University College Cork, Wilton, Cork, T12E8YV Ireland; 3 College of Medicine and Health, Erinville Hospital Western Road University College Cork Cork, 3rd Floor, Cork, T12EKDO Ireland; 4 Centre for Health Policy and Management, School of Medicine, Trinity College Dublin, 3-4 Foster Place, Dublin 2, D02 PN40 Ireland

**Keywords:** Oral health policy, Public dental systems, Universal healthcare, Oral health policy analysis, Reform

## Abstract

**Background:**

Calls are emerging for oral health system reform under the Universal Healthcare (UHC) domain, while internationally there is an absence of political priority for oral health. In the Republic of Ireland there is very limited coverage of oral healthcare for the whole population. ‘Smile agus Sláinte’ Ireland’s oral health policy published in 2019, represents the first change to national policy in over 25 years.

**Methods:**

This research examined the key factors influencing oral health policy, development, and implementation in Ireland during the period 1994–2021. A case study approach was adopted with two strands of data collection: documentary analysis and semi-structured interviews with elite participants. Analysis was guided by Howlett’s five stream framework.

**Results:**

Ireland shares the international experience of oral health having very low political priority. This has perpetuated unequal access to public dental services for children and special needs populations while austerity measures applied to adult schemes resulted in increased unmet need with no universal coverage for dental care. The only area where there is political interest in oral health is orthodontic care. This low political priority combined with a lack of actor power in national leadership positions in the Department of Health and Health Service Executive has contributed to successive non-implementation of oral health policy recommendations. This is most evident in the failure to publish the Draft National Oral Health Policy in 2009. The research finds a failure to adequately engage with key stakeholders, particularly the dental profession in the development of the 2019 policy. All these weaknesses have been exacerbated by the COVID-19 pandemic.

**Conclusions:**

Ireland’s new oral health policy, ‘Smile agus Sláinte’, presents an opportunity for the provision of much needed public dental services. However, successful reform will require strong political will and collaboration with dental leadership to provide advocacy at national level. Global calls to incorporate oral health into the UHC agenda and an agreed political consensus for UHC in Ireland may provide an opportunity for change. Genuine engagement of all stakeholders to develop an implementation strategy is necessary to harness this potential window of opportunity for oral health system reform.

## Background

Most oral health conditions are largely preventable [1]. Yet oral diseases affect 3.5 billion people worldwide with untreated dental caries of permanent teeth the most prevalent condition [2, 3]. The burden of oral disease is hallmarked by significant inequality, disproportionately affecting marginalised populations and low socio-economic groups [4]. As such access to prevention, early diagnosis and treatment of oral diseases is “unattainable for millions of people”[2].

The main strategy for mitigating inequalities impacting access to oral health services is implementing oral health policies [5]. However, oral diseases are a neglected issue often failing to receive priority in broader health and public policies [6, 7], “isolated and marginalised” from health policy developments [4]. A lack of political commitment and resources along with the failure to prioritise disease prevention are contributory factors [2]. Responding to the global “state of crisis” in oral health requires policy change from traditional clinical approaches particularly to align with the Universal Healthcare (UHC) agenda [2, 8–10].

The Republic of Ireland (Ireland) is a small country in Western Europe with a population of 5.01 million people. The Irish general health system is “complex” with access to care often based on the ability to pay rather than medical need [12].The latest available data from the Organisation for Economic Co-operation and Development (OECD) shows Government financing as a proportion of total health expenditure was 75% in 2019, a comparatively low figure compared to the EU average of 80% [13]. For oral healthcare in Ireland, delivery is via a public/private mix of service provision [14]. However, gaps in publicly funded oral healthcare for significant parts of the population mean high out of pocket payments that may cause financial hardship [12]. In contrast to the general health system, it is estimated that almost two thirds of all dental expenditure in Ireland is privately financed, the majority of which relates to direct out of pocket payments. Furthermore, while almost half the Irish population voluntarily purchase private health insurance (PHI), this is primarily to facilitate quicker access to planned hospital treatment or private medical care. PHI offers only “limited coverage” of dental care [12, 14].

Leadership, governance and policy direction for the health system is provided by the Department of Health (DoH)[16]. The Health Service Executive (HSE) established in 2005 is a national government agency under the aegis of the DoH responsible for the management and delivery of health and social care services [17]. Following a decade of economic prosperity, Ireland experienced one of the most severe economic crises of any OECD country between 2008 and 2014 [18]. During this time, austerity measures which had a lasting impact on the health system, including limiting access to dental services, were introduced [12, 19]. However, by 2017, Ireland was once again experiencing high levels of economic growth [20].

Ireland is the only western European country without universal primary healthcare and despite many years of reform there is a consensus that the health system is inequitable and underperforming [17]. Significant gaps in universal health coverage are a key consequence of Irelands complex system and as mentioned, access to publicly financed dental care is particularly poor, at just one third of all oral health expenditure [12, 13]. In 2017, the Irish Government committed to a 10-year plan developed by a cross-political party committee, toward the delivery of a universal single-tier health service where individuals are treated solely based on need, known as ‘Sláintecare’ [20].

In March 2019, Irelands national oral health policy known as ‘Smile agus Sláinte’, was published [21]. Its key goals are to support individuals to achieve their best oral health and reduce inequalities across the population, while the fluoridation of piped water supplies is supported as an oral health protection policy [21]. ‘Smile agus Sláinte’ aims to align with the “same ideals” as the proposed universal health service, ‘Sláintecare’, however it describes the provision of ‘preventive bundles’ of care delivered at various ages throughout the life course [21]. The planned large scale reform of the public dental system outlined in this policy is considered “challenging” by policymakers [21].

Health policy analysis is crucial to health reform [22]. It is useful retrospectively and prospectively to understand past policy successes and failures and in planning for future policy implementation [23]. Previous research illustrates that with respect to oral health policy development, a better understanding of the complex interactions between context, policy processes and actors can enable the design of more responsive policies [24]. In particular the broader socio-political contexts in which actors develop oral health policies can facilitate and/or constrain actors roles and policy processes [24]. There is limited international research analysing oral health policy [24–27] and to the best of our knowledge, none in the Irish setting.

The aim of this research is to generate an in-depth understanding of the key factors that impeded or promoted the development and implementation of oral health policy in Ireland during the period 1994 to 2021.

## Methods

To facilitate a comprehensive analysis, a case study approach was adopted [28]. Case studies are considered beneficial for “in-depth, multi-faceted explorations of complex issues in their real-life settings”[29]. To strengthen study validity and generate a thorough understanding of the case, the use of multiple data sources is advised [30]. Two strands of data collection and triangulation were thus undertaken—a documentary analysis and semi-structured in-depth interviews with ‘elites’ [31].

### Documents

A documentary analysis examined the relevant reports, policies, service documents and other literature developed at local, regional, and national level from 1994 to 2021. These dates were chosen as 1994 is the date of Irelands’ first national health policy which included a ‘Dental Health Action Plan’ publication [32] and Ireland’s recent national oral health policy was published in 2019 [21]. The study was expanded to include the impact of the COVID-19 pandemic to the time of writing in July 2021. Two documents published prior to 1994 were included in the dataset as their contents were deemed important to the analysis of the ‘Dental Health Action Plan’ [33, 34].

Documents reviewed were identified by performing searches of:

1. Government websites particularly the website of the Department of Health, for policies, reports, and guidelines.
2. The Lenus database, the Health Research Repository of the Irish health service.
3. Records of parliamentary debates, governmental committee meetings and parliamentary questions on the ‘Oireachtas’ website.

A total of 125 documents were identified, reviewed, and mapped over a 27-year timeline. The documents analysed included health policy documents (*n* = 14), commissioned reports (*n* = 18) and health service documents (*n* = 19). A detailed overview of the dataset including the quantity and nature of included documents is presented in Table 1.

**Table 1:** An overview of documents incorporated in analysis

Document type	Number (n)
Policy documents^1^	14
Commissioned reports^2^	18
Health strategies^3^	9
Health service documents^4^	19
Non-governmental reports/strategies^5^	7
Legislation^6^	5
Reports of epidemiological surveys^7^	7
Parliamentary papers^8^	
Parliamentary questions	33
Presentations to parliamentary committees	4
Clinical guidelines^9^	4
Academic and grey literature^10^	5
Total	*n* = 125

^1^Policy document: any publication with stated governmental policy positions including general and oral health policies, published and unpublished, along with associated policy frameworks, implementation, and action plans

^2^Commissioned report: any document commissioned by government including reports from external bodies and institutions along with inter departmental requests

^3^Health strategy: any document detailing steps and strategic intent by government, but not as actual government policy, for example health department statements of strategy

^4^Health service documents: any document outlining specific health and social service delivery, objectives, resources, actions, and outputs, primarily including HSE National Service plans

^5^Non-governmental reports/strategies: any relevant report/strategy published by a non-government affiliated organisation

^6^Legislation: relevant legal acts and regulations

^7^Parliamentary question: any relevant question raised by a member of parliament provided with an oral or written response

^8^Presentation to parliamentary committee: any evidence verbal or written presented to relevant governmental committees

^9^Clinical guidelines: a series of evidence-based guidelines for those planning and providing dental services for children in Ireland

^10^Academic and grey literature: relevant academic and grey literature used to enhance contextual nuance of included documents

### Elite interviews

The outcomes of the documentary analysis enabled the categorisation of key stakeholder groups across the oral health policy (OHP) landscape in Ireland: The Health Service Executive, private dental practitioners, academics, and policymakers including the Department of Health. In-depth elite interviews were then undertaken with actors purposely identified as representative of each stakeholder group. Participants were primarily identified via the documentary analysis, supplemented by purposive sampling based on the recommendation of participants and the researchers’ knowledge of actors closely engaged in OHP and/or OHP development. A piloted interview guide informed by the documentary analysis was employed during interviews and the list of participants was continually reviewed throughout the data collection process.

In all fifteen participants (n = 15) were invited to interview, with thirteen (n = 13) consenting to participate: four representatives of the HSE (n = 4), two representatives from private dental practitioners (n = 2), five academics (n = 5) and two policymakers (n = 2). Most respondents (n = 11) were trained dental practitioners, and all had a detailed knowledge of OHP and/or OHP development. All but one of the interviews were conducted via teleconferencing facilities between October 2020 and March 2021 in accordance with COVID-19 related public health guidelines.

### Data analysis

**Fig. 1:**
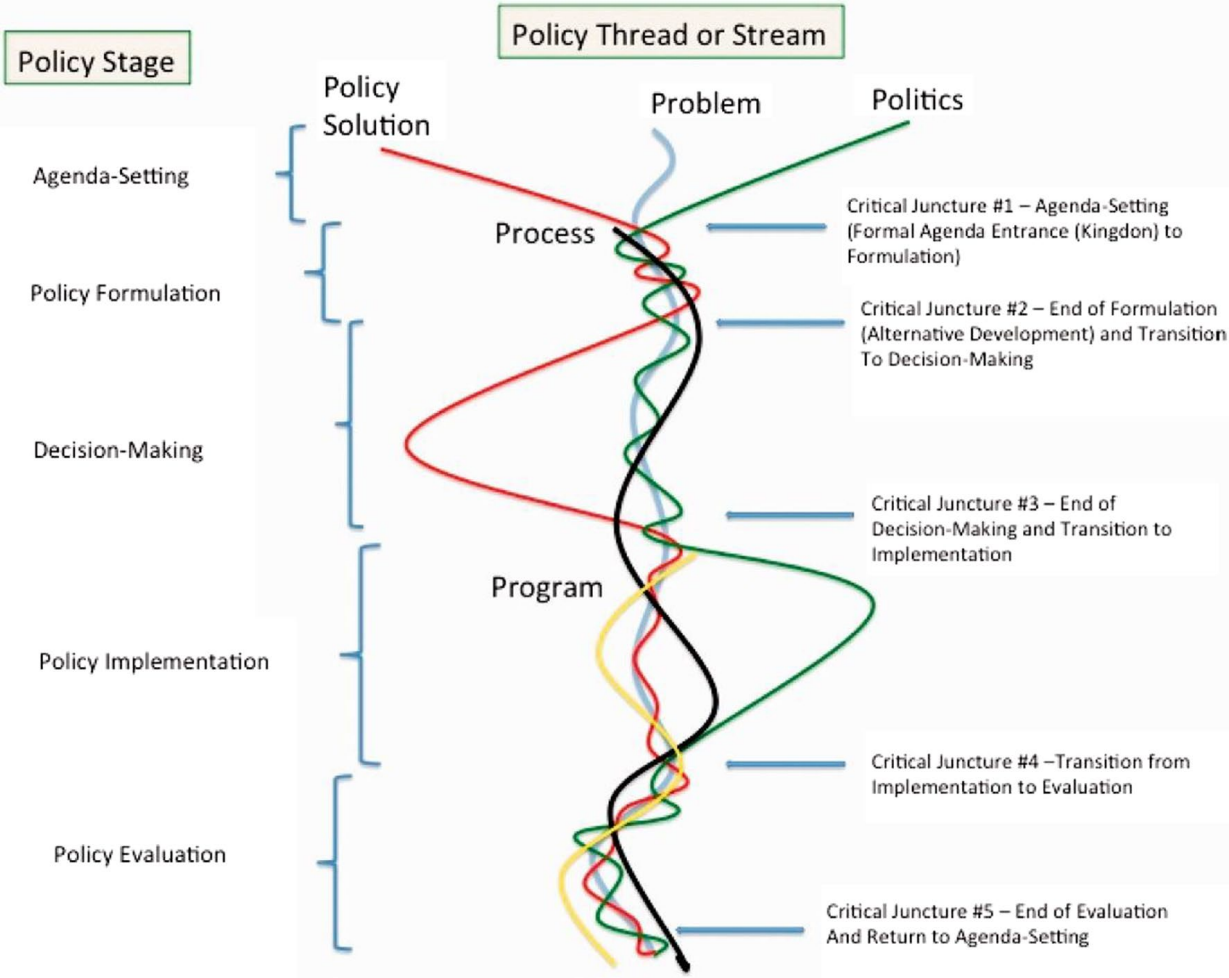
Figure Howlett’s five stream model. *Source*: Howlett et al. 2019

This research followed best practice guidance for health policy analysis by rooting the analysis in an existing framework [23]. The ‘Five stream framework’ by Howlett et al. [35] was chosen for analysis due its comprehensive, wide raging coverage and ability to capture a multitude of elements in complex policy processes. The five streams (Fig. 1) are the problem, policy, process, politics and programme streams [35]:

1. *Problem* to the framing and articulation of policy problems.
2. *Policy* refers to the formulation of policies, policy alternatives and instruments.
3. *Process* refers to the main tasks and events which lead to policy outputs.
4. *Politics* refers to the politically active policy actors.
5. *Programme* refers to the management of implementation [35].

Qualitative data analysis software (NVivo 12) was used and followed a directed content analysis approach [36] guided by the structured framework. Initially, the contents of each document were mapped onto an MS Excel spreadsheet using a range of column headings. These headings included title, author, year, resource type, source/location, political landscape, and content. A range of key concepts were then identified as coding categories using the framework as guidance before operational definitions for each category were determined. The operational definitions derived from the framework in addition to the documents included in the dataset were agreed by all authors. Interviews were transcribed verbatim and coded.

All transcripts and outcomes from the documentary analysis were examined together and coded using NVivo. The predetermined categories derived from all five streams were applied across all documents and interviews where relevant and applicable, with any remaining text coded with another label and captured.

Throughout the data analysis process, the findings from interviews were continually triangulated with the outcomes from the documentary analysis following best practice for policy analysis. The data generated from the documentary analyses provided an adequate description of the technical content of key policies and literature [37]. While elite interviews were used to elaborate on the documents’ findings in a more in-depth manner particularly to understand why specific policy choices were made or not made [31]. Emerging findings were discussed in structured meetings with all authors who have extensive dental research or policy analysis experience.

### Ethical considerations

Ethical approval for the study was obtained from the Clinical Research Ethics Committee of the Cork Teaching Hospitals at University College Cork (CREC) (Reference number: ECM 4 (J)12/11/2019). All methods were carried out in accordance with the guidelines and regulations determined by the CREC. At all times, ethical considerations including ensuring confidentiality, obtaining informed consent, and preserving anonymity were adhered to. Informed consent to participate was granted by each participant.

## Results

The findings are presented utilising Howlett’s streams (Fig. 1) and draw on findings from two datasets, a documentary analysis and interviews, triangulated to build an overall perspective.

### The problem stream

The documentary analysis found that Ireland has very restricted dental coverage for the whole population [12]. Publicly funded dental services are delivered via three schemes: (i) the Public Dental Service (PDS), (ii) the Dental Treatment Services Scheme (DTSS) and (iii) the Dental Treatment Benefit Scheme (DTBS) (Table 3) [14]. A significant proportion of the population (20% in 2016) are not eligible for any scheme and must either purchase private dental insurance or pay out of pocket for private dental care [12, 14]. Those patients may claim back fees through tax relief up to a maximum of 20% of the treatment cost for certain non-routine procedures [15].

The ‘Public Dental Service’ (PDS) is the only state funded dental care for children and special needs populations, delivered by salaried dentists directly employed by the state [21]. The PDS aims to “target” children for dental care in “three designated classes in primary and secondary schools”[32]. According to documents analysed, the practice of targeting first emerged as a rationing mechanism in response to economic constraints in the 1980s [33] by targeting two age ranges associated with the eruption of permanent teeth (i.e., ages 7–8 and 11–12) while emphasising the need for improved orthodontic services[33, 34].

There is no further access to State supported dental services for any child under age 16, except in the event of an emergency [21]. The PDS has suffered significantly from austerity measures first introduced in 2009 with a 20% reduction in staffing levels [38, 39]. It was further impacted by the COVID-19 pandemic with 23% of dental professionals redeployed to COVID-19 related work [40]. Access to care is determined by age rather than need and despite recommendations to the contrary [41] it remains the policy guidance under which dental services for children and special needs groups operate in 2021.

Inequalities with respect to dental care for children were highlighted in the documentary analysis. For example a report commissioned by the DoH in 2002 described elements of the system as a “disgrace” [42]. This remained an issue of common concern amongst interviewees when considering public dental services for children:

Usually, it’s one, maybe two (primary school) classes you’re seeing properly…it’s not kids from whatever zero to sixteen. They say every child under the age of sixteen. That’s not true at all. (P2).^1^

([The Public Dental Service]) just papers over the cracks…different regions have different resources…so if you live in one place two (primary school) classes might be seen, but drive down the road and you could have one, or none, same with special needs…^2^ (P3)

The Gelbier report [42] also advocated for improved services for special needs groups: “it is essential for this report to make the strongest of recommendations on behalf of special needs child and adult patients…a duty of care for those who cannot look after themselves” [42]. It is estimated that 95% of the activity of the Public Dental System pertains to services provided to children, with just 5% of care going to patients in vulnerable groups [15]. While the reports of epidemiological studies that emerged from the documentary analysis found that permanent teeth were more frequently extracted in Irish children with a disability than the general population [43, 44]. The difficulties prevailing in special care dentistry also featured prominently in interviews:

I feel extremely…very, very strongly about the…failure to invest in special care dentistry…. (P4)

The problems for special needs groups…It’s really upsetting…it’s heart breaking…. (P6)

An unpublished report identified during this research which was commissioned by the Department of Health in 2009 found a “disinterest’ in the public dental system by senior HSE management had a "demoralising effect on service providers” [45]. These sentiments were echoed by interviewees familiar with its operation:

We just do what we are told…stagger on…(P2) and …it’s like military rule… (P1)

Insufficient attention is paid to any of the good outcomes…it’s been next to impossible to get any attention from senior people in the HSE or the Department of Health…. (P4)

Dental services for adults are provided through the Dental Treatment Services Scheme (DTSS) and the Dental Treatment Benefit Scheme (DTBS) (Table 3) where dentists are contracted by the state to provide care in privately owned practices [21]. Both schemes were among the first health budgets to face cuts and suffered severely because of austerity measures introduced in 2010 [12]. This described by an interviewee: “The state basically ditched large swaths of the dental profession…” (P8).

Funding of the DTSS scheme was reduced from €62 to €10 million between 2010 and 2015 [20]. Dental benefits were limited to an examination and two emergency restorations with any other care subject to approval from the local HSE health manager. However, coverage was provided for unlimited dental extractions [12]. The restrictions in preventive and restorative oral health care while funding remained uninhibited for dental extractions is described by an interviewee: “…Talk about equity and fairness…how could we do that? It’s probably driven the level of extractions up…its almost medieval…” (P6)

The DTBS scheme was limited to examination only until provision for a scale and polished was reintroduced in 2017 [12]. According to an interviewee: "The darkest day in dentistry was when the budget for 2010 was announced…in dentistry it was treatment service to the patient that was curtailed, that didn’t happen for other services like GPs or pharmacy…" (P4).

The documentary analysis concluded the changes introduced had a significant impact resulting in unmet need for dental care tripling between 2008 and 2012 [12]. Despite calls to reverse the austerity measures, the funding cuts introduced in 2010 still remain in place in 2021 [12, 20]. The problems occurring in the DTSS Scheme were of concern for many interviewees: "The DTSS has been decimated, the system is basically not functioning for…10 years, dentists are withdrawing because it’s no longer lucrative, patients don’t even know what’s available to them…it is entirely dysfunctional…the Department [of Health] would prefer if everyone was edentulous…" (P13).

### The policy stream

#### 1994—the dental health action plan

The first Irish health policy ‘Shaping a Healthier Future: A Strategy for Effective Healthcare in the 1990s’ was published in 1994 [46]. It was a policy document which included a series of action plans including a Dental Health Action Plan’ (DHAP). This was the ‘first clear statement on the aims and health objectives of the dental services in Irish history’ [32] (Table 2).

**Table 2:** Irish National Oral Health Policies 1994–2019

Title	Year	Document	Key findings	Main recommendations
The Dental Health Action Plan	1994	An action plan published as part of the national health policy-Shaping a Healthier Future: A strategy for effective healthcare in the 1990s The current policy guidance for all publicly operated dental schemes in Ireland in 2021	No systematically organised care for pre-school children Care for primary school children is uneven Poorer oral health in medical card holders Increased demand for orthodontics Poor services for those with additional needs	Goals for reduced dental decay across population by 2000 Continue & increase fluoride use (water & toothpaste) with support from industry Consolidate & extend targeted school dental services Extend eligibility for care to age 17 Care for adult medical holders through new scheme (DTSS)
Draft National Oral Health Policy	2009	Unpublished by the Department of Health	Oral health has improved since the introduction of the Dental Health Action Plan for some groups However improved oral health not observed in medical card holders or children under-age-5 Mismatch between peaks of childhood caries and age range the PDS focuses on Deficiencies in care for those with disabilities, special needs and elderly in long stay institutions Overlaps between DTSS and DTBS schemes with co-ordination mechanisms lacking to ensure cost effectiveness and equity	Administration of DTSS and DTBS merged to enhance equity and reduce administration costs PDS: Refocus resources to address need, improve data management andmonitoring of outcomes with a senior clinical manager to head service Identify people with special needs and social disadvantage for appropriate oral healthcare Enhance the regulatory regime governing oral healthcare Recognise specialty of Special Needs Dentistry immediately. Progress specialisation in Public Health Dentistry following new arrangements in PDS Allow dental hygienists operate independently Continue water fluoridation Integrate oral health promotion with general health promotion
Smile agus Sláinte	2019	National Oral Health Policy No implementation strategy published by the end of 2021	Inequalities in service provision for very young, vulnerable, those with disabilities and elderly Decline in dental decay since 1980s 30% of 5-year-olds, 40% of 12-year-olds have dental decay Symptom led use of services for children under age 6 Poor uptake of oral services by the elderly, rising incidence of head and neck cancer in older groups Three times more dental practices owned by private practitioners than HSE premises Current service not designed to deliver prevention and is not a comprehensive primary care service	41 actions, DoH leading transformation over 8-years Primary oral healthcare approach with Sláintecare ideals Reorient service into 3 streams: primary care (independent practitioners), community care (HSE salaried practitioners) and advanced oral healthcare centres Care delivered through packages, defined by age Care for adults via medical card only All access will begin at primary oral healthcare, referral to secondary and tertiary care for one off treatment Upskilling of HSE practitioners required National oral health evaluation programme with WHO indicators Oral health promotion programmes for disadvantaged and non-fluoridated areas Topical fluoridation programmes for under-6’s not supported

The DHAP was viewed by most interviewees as generating improvements in public dental services: “The dental health action plan did make progress, it put some adult services out there, some special care services…it did increase the number of teams.” (P4) While its dental public health approach was welcomed: “It had a vision, a philosophy underpinning it…a recognition of inequalities…progress was made” (P7). However, there was a failure to maintain progress: “The platform was reached. And then very little was built on that platform (P4)”. There were difficulties with workforce distribution: “The poorer areas where it was difficult to recruit dentists were getting the minimum…” (P11) and according to documents, concerns were raised regarding management and probity assurance in DTSS Scheme [45, 47].

#### 2001—quality and fairness, a health system for you

In 2001, the national health policy ‘Qualify and Fairness, A Health System for you’ was published [48]. It defined ‘Schools Dental Services’ as free of charge for all and specified a series of actions including: (i) a review of the DHAP (ii) new goals for oral health (iii) the expansion of specialist training programmes and (iv) more use of private sector orthodontists. An accompanying ‘Primary Care Strategy’ [49] assigned dentists to the ‘Primary Care Network’. It recognised the ‘particular implications’ for dentists delivering public services in privately owned premises however no solution was proposed, nor was there any inclusion for dentists in human resource planning, education or training [49].

#### 2002—the forum on fluoridation: a policy review

Community Water Fluoridation (CWF) was adopted as health policy in Ireland in 1964 [50]. Responding to public concerns regarding patient safety a major policy review of CWF was undertaken in 2002 [51]. The review supported CWF as safe and effective while proposing a fluoride reduction in water supplies and toothbrushing recommendations in response to increasing levels of fluorosis [51].

#### 2009—unpublished national oral health policy

During this research, a Draft National Oral Health Policy’ first announced by the Minister for Health in 2007 and completed in 2009 was identified via both strands of data collected [52] (Table 2). The policy remains unpublished, with an unpublished copy obtained by the lead researcher.

This document acknowledged the “apparent mismatch between peaks of childhood caries and the age range on which the PDS focuses” and the “deficiencies in care” offered to special needs groups [52]. The policy is referenced in the HSE National Service Plan 2008 [53] but according to one interviewee engaged in its development “it never saw the light of day” (P6). Another interviewee stated: “There was a lot of buy in…a lot for everybody…but…it never went through…and then the economic collapse took it off the table” (P12).

In a response to a parliamentary question in 2009, the then Minister for Health Mary Harney stated: “The National Oral Health Policy has to be reconsidered in light of the current position of the public finances” [54]. By 2011, the HSE National Service Plan signalled the end of the unpublished draft policy stating that a “new policy framework will be developed” [55].

#### 2017—Sláintecare

Oral health did not feature in any other national health policy document between 2001 and 2017 except for one mention of community water fluoridation [56]. In 2017, the Sláintecare report, a 10-year plan for health reform was published by a cross-political party committee following a 2016 Programme for Government Commitment. It recommends dental care to fall under the remit of UHC and urges reinstatement of the pre-economic crisis DTSS budget as a “short term” measure [20]. A “universal comprehensive package of care” following publication of a new oral health policy in 2017 is recommended. In 2018, it was adopted as government policy when the ‘Sláintecare Implementation Strategy’ was published with dentists referenced as community-based care, but no further detail on oral healthcare was provided [57]. Following this, the first ‘Sláintecare Action Plan’ was published in 2019 with no mention of oral health [58].

In 2018, ‘First 5: A Whole-of-Government Strategy for Babies, Young Children and their Families 2019–2028’ was released “in line with Sláintecare” [59]. It recommends the introduction of “a universal dental health package for children under six, supported by a screening/surveillance programme to target key ages and vulnerable groups” [59].

#### 2019—Smile agus Sláinte

‘Smile agus Sláinte’ represents the first update to national oral health guidance since 1994. The need for a new policy is attributed to changing population demographics, technologies and treatment requirements [21]. It outlines a plan for dental coverage through bundles of care for those under-age-16, vulnerable groups and medical card holders delivered at various stages throughout the life-course. Out of pocket charges are to continue for the remainder of the population (Table 2) [21].

Its publication in 2019 was broadly welcomed by interviewees: “It’s a well-intentioned, well-researched, well-structured document” (P1) with the emphasis on prevention and integration highlighted: “…the positives of the policy are…more treatment for medical cardholders and more preventive treatment…those are huge positives (P3)” and “…that integrated system…everybody can endorse (P13)”.

‘Smile agus Sláinte’ proposes the reorientation of dental services for children from salaried services delivered in State owned premises to individual dentists contracted to provide treatment in “primary oral healthcare settings” [21]. This significant change was highlighted by interviewees: “The pivoting of the profession is very ambitious…it’s a seismic task” (P4 ). While the “primary care approach” was largely supported: “Its more sustainable long term… I think that the Public Dental Service (Table 3) needs to… become what the policy envisages, a high-risk service” (P1).

**Table 3:** An overview of publicily funded oral healthcare in Ireland in 2021

Dental scheme	Patient group covered	Type of oral health care covered
Public Dental Service	Children (0–16 years) There are 1.07 m children under age 16 in Ireland 22% of population Some adult populations with special needs	‘Emergency’ dental care only Comprehensive treatment entailing preventive and restorative care (including orthodontic treatment under strict qualification criteria) Two age groups only (ages 7–8 and ages 11–12) Eligibility does not equate to accessibility Service provision varies across the country Patients are targeted by age not clinical risk
Dental Treatment Services Scheme (DTSS)	Category I Medical Card holders Eligibility for a Medical Card is generally based on an income means test Covers 32% of the population 24% of eligible persons utilised scheme in 2019	One oral exam per annum, two fillings per annum and unlimited dental extractions Prior approval can be sought from a local HSE health manager for additional treatment including: More than 2 fillings, full or partial dentures, anterior root canals and for certain high risk groups, periodontal care
Dental Treatment Benefit Scheme (DTBS)	Social insurance contributors for three years (PRSI) Up to 2.2 m contributors insured in 2018 at a cost of €50 m In 2018 1.25 million claims were approved	Oral examination and one scale and polish (to the value of €42) annually

As of April 2021, there were 3320 registered Dentists in Ireland of which an estimated 2000 are actively practising with 316 salaried dentists directly employed by the HSE in the Public Dental Service

The identification and management of vulnerable children was a concern raised by some: “We need a very clear safety system for children who may need a little bit extra”(P10). Along with the acceptability of the proposal to the dental profession: “They have assumed that there will be no kickback from the community dentists but not everybody wants to see special care adult patients…if a person isn’t good with children…they shouldn’t be seeing children” (P10). There were also significant concerns regarding the workforce skill mix available: “The whole area of higher end training and specialization necessary…was deliberately underplayed.” (P4).

### The politics stream

The strongest and most persistent finding emerging from both the interviews and documentary analysis is the low political interest in oral health in Ireland. This is surmised by an interviewee: “Oh god there’s absolutely no [political] interest, none, not at all, they’ve bigger fish to fry…dentistry isn’t a priority”. (P2).

From the documentary analysis it is evidenced by the absence of oral health content in national health policies [48, 49, 56, 60, 61]. Further reinforced by the failure to implement most recommendations from commissioned reports, research, guidelines, and policies over two decades [41–44, 52, 62–64].

Additionally, according to an analysis of reports commissioned by the Department of Health, the absence of dental leadership in the health Department and an advocate for oral health in national management in the HSE resulted in a failure to promote oral health and drive policy nationally [45, 47] (Table 4). An interviewee stated: “There was a lot of messing, the role of the chief dental officer lay fallow for a while…in the department there was a lack of willingness, a lack of lobbying, a lack of leadership” (P6). The political disinterest following the impact of austerity measures was also noted: “[Public Dental] Clinics were closed, and nobody complained. Nobody missed them. That's frightening.” (P1).

**Table 4:** An overview of key political actors in Irish Oral Health Policy landscape: 1994–2021

Year	Health Minister Political parties in government with ministerial party in bold	Dental presence in Department of Health
1993–1994	Minister Brendan Howlin *Fianna Fáil/****Labour***	Assistant Chief Dental Officer
1994	Minister Michael Woods ***Fianna Fáil****/Labour*	Assistant Chief Dental Officer
1994–1997	Minister Michael Noonan ***Fine Gael****/Labour/Democratic Left*	Assistant Chief Dental Officer 1994–1995) Appointed ‘Chief’ Dental Officer (1996)
1997–2000	Minister Brian Cowen ***Fianna Fáil****/Progressive Democrats*	Chief Dental Officer
2000–2004	Minister Michael Martin ***Fianna Fáil****/Progressive Democrats*	Chief Dental Officer
2004–2011	Minister Mary Harney *Fianna Fáil/****Progressive Democrats***	No Chief Dental Officer
2011	Minister Mary Coughlan ***Fianna Fáil****/Progressive Democrats*	No Chief Dental Officer
2011–2014	Minister James Reilly ***Fine Gael****/Labour*	No Chief Dental Officer 2011–2013 HSE National Oral Health Lead seconded 2 days a week as Chief Dental Officer in 2013
2014–2016	Minister Leo Varadkar ***Fine Gael****/Labour*	Chief Dental Officer (Part-time)
2016–2020	Minister Simon Harris ***Fine Gael****/Fianna Fáil*	Chef Dental Officer
2020-Present	Minister Stephen Donnelly (incumbent) *Fine Gael/****Fianna Fáil***	Chief Dental Officer

Fianna Fáil (FF): Dominant from 1997–2007, nationalist and conservative, the largest and oldest political party in Ireland at that time

Fine Gael (FG): Traditional rival of Fianna Fáil, a party of the centre right, liberal and conservative. Both FF and FG have seen their once broad popularity decline in recent years

Progressive Democrats (PDs): Formed by a group split from FF, pursued economically liberal policies with a strong low-tax, pro-business focus. Had considerable influence over government policies particularly economic and health policy

Labour: A party of the centre left described as a democratic socialist party

The exception is the high political priority attributed to orthodontics. In times of scarce resources orthodontics received precedence. This was criticised in a report commissioned by the DoH in 2002 [42]: “One wonders about the logic of providing orthodontics at a time when there appears to have been many other needs to be satisfied (e.g. tooth decay in young children)”[42]. An analysis of documents found that in 2015, as austerity measures remained in place, additional public funds were allocated to orthodontic care [65].While in April 2021, politicians raised concerns regarding orthodontic waiting times during a governmental committee debate that was established to consider the difficulties faced by low income adults accessing publicly funded dental care. [66].

This priority was reflected on by an interviewee: “Orthodontics will always have a conversation because they're the articulate people…When you represent and advocate on behalf of the vulnerable adult, and the child, it falls on a political deaf ear. And that's the fact” (P10). and another: “Politically what we know is that the one important thing is orthodontics…the middle-class voter wants free orthodontics for their child and so that's what the politicians go for” (P6).

Table 4 details Irish health ministers, their political affiliation along with the presence of expert dental opinion advising government during the study period. This overview illustrates a predominance of centre right politicians responsible for health in Ireland. The exception is a centre-left minister in 1994 which coincided with political priority for oral health policy followed by policy implementation and subsequent public dental system reform.

### The process stream

**Fig. 2:**
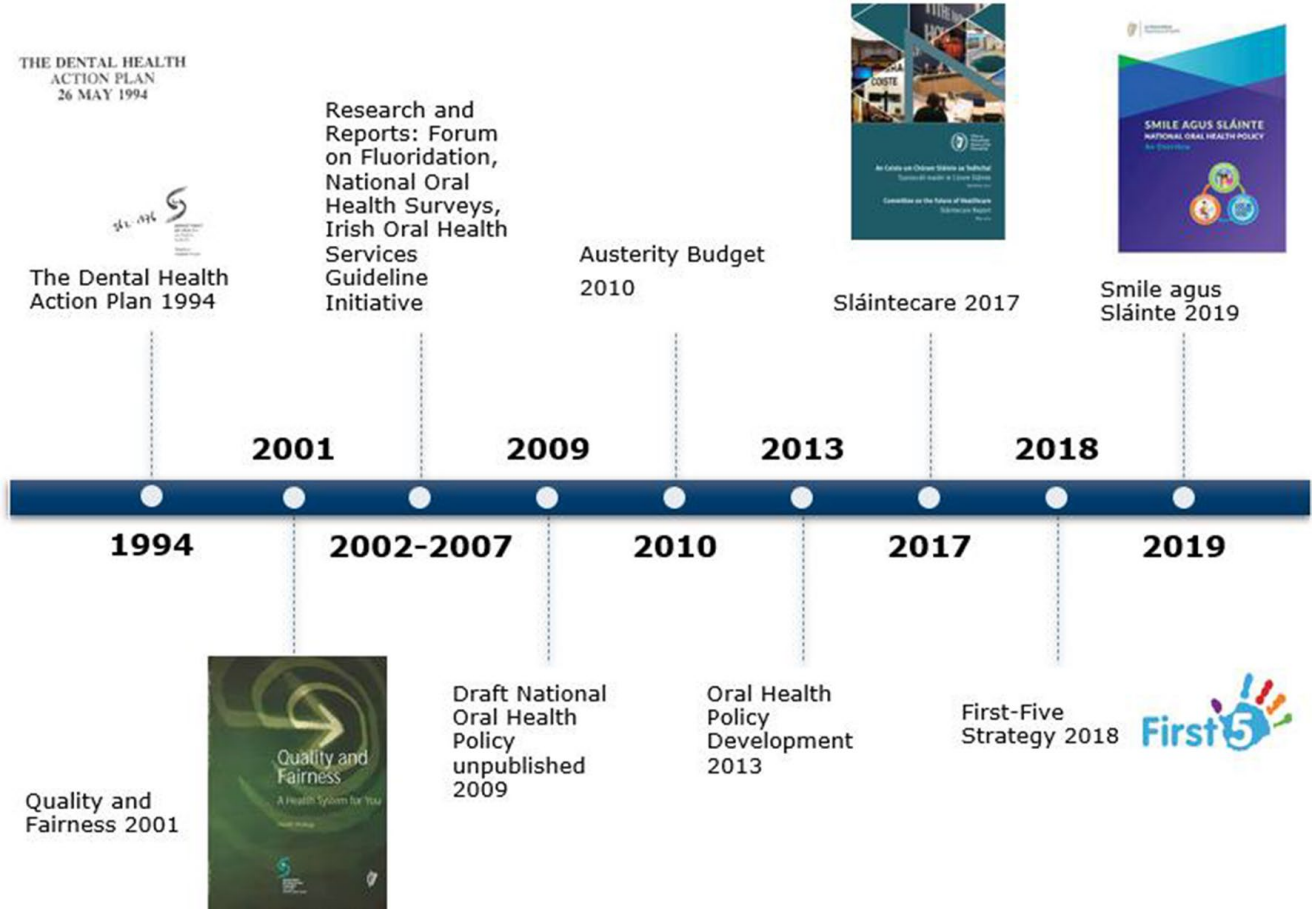
Timeline of key oral health policy milestones in Ireland 1994–2021

The process stream is concerned with policy development [35]. For this research, the development of the ‘Dental Health Action Plan’ (DHAP) [32], the unpublished Draft National Oral Health Policy in 2009 and ‘Smile agus Sláinte’ [21] are most relevant. An overview of the key policy milestones for oral health in Ireland is illustrated in Fig. 2.

### Development of the DHAP

A DoH report in 1988 found the public dental service was failing in its statutory duty to provide dental care for eligible adults [33]. By 1990, public anger arising from media reporting of medical card holders with missing teeth drove oral health onto the political agenda [42]. According to an interviewee: “What was…appearing in the media was that the elderly were complaining they couldn't get dentures…And…the big complaint about orthodontics, you couldn't get the kids teeth straight…So those two things were piling in on top of politicians.” (P11).

In the early 1990’s the need for a new health policy for Ireland was emerging [67]: “…not only were the department [of health] devoting attention to teeth but an overall health strategy….so the plan for the dental services fitted” (P11).

At the same time, findings emerged from a national survey of adult oral health demonstrating that lower socioeconomic groups had poorer oral health and greater treatment needs than the general population [68]. This is described by an interviewee:

Everyone knew medical card holders couldn’t get a service…it was just…inequitable…the hard evidence really showed it… (P6)

According to both strands of data in this research, the media spotlighting oral health inequalities at the same time a national health policy was under development combined with the outcomes from the national adults survey provided an opportunity for oral health to briefly garner political support. The ‘Dental Health Action Plan’ was subsequently published in 1994 [32].

This is described by an interviewee: “Talk about right party, right place, right time…The Secretary General, the Minister, everybody was on the same page. It was a transformative moment. It was presented as such, it was believed as such, this just wasn't a one-off kind of initiative.” (P11).

An outcome of the DHAP was the establishment of the DTSS scheme following negotiations with the dental professional representative group, the Irish Dental Association (IDA) [32]. This success is attributed to shared leadership, collaboration, and effective communication by interviewees:

The key thing was…leadership, enlightened leadership on all sides, totally committed people with very good communication skills…who saw they were doing something good. (P11)

We had really good representation in the Department [of Health] at the time…and the civil servants were skilled. (P6)

### Development of the unpublished Draft Oral Health Policy

According to an analysis of parliamentary documents, an investment in dental research of £1 m along with an expenditure of £2.34 m on commissioned reports between 1999 and 2007 brought oral health back onto the political radar [69]. On 18th October 2007, the Minister for Health launched the development of a new Oral Health Policy [70]. According to an interviewee, a report advocating for reform of the Irish dental profession by the competition authority [71] and investment in dental research instigated policy development: “The drivers were…the Competition Authority report…the Department [of Health] had spent a lot of money on research…and the legislation, anybody could see the dentist act needed to be updated” (P12).

Documents identified during the course of this research reveal that a consultative and core policy development group was formed in the DoH in 2007 [52]. Two studies were carried out, an analysis of the Public Dental System [45] and an economic analysis on the possible integration of the state dental schemes (the DTSS and DTBS) with the findings presented in March 2008. The draft National Oral Health Policy was developed by 2009 but remains unpublished.

### Development of Smile agus Sláinte

According to the documentary analysis, the development of ‘Smile agus Sláinte’ commenced in 2013 when the HSE National Oral Health Lead was released to the DoH for two days each week to undertake the functions of Chief Dental Officer. One of their primary responsibilities was the development of a new oral health policy [72]. The policy document details the development process including a series of working groups with oral health professionals, an Academic Reference Group, an accompanying ‘Practitioners Reference Group’ along with an ‘Independent Panel’ [21]. According to an interviewee:

It started well…the policy was announced, feedback was encouraged…but as the years went on the amount of feedback and communication diminished. (P7)

Findings from both documents and interviews highlight that progress was slow – the policy development process started in 2013 and the policy was published in 2019 [21, 72]. A key finding from most interviews carried out for this research is a perceived failure to engage with all relevant stakeholders particularly representatives of the HSE and private dental practitioners as the policy was being developed. This is described by an interviewee engaged in policy development: “You needed to bring the dental professional, along with you” (P5).

…the most important people in the process weren't really considered at all. (P13)

Interviewees also raised queries about the secrecy and time taken during the policy process: “…hush hush and top secret… It was going in the right direction but too secretive…we were waiting twenty years…” (P2). While interviewees participating in policy development initiatives queried the extent to which their inputs were considered: “…the final document, I can't see any evidence of…the group that is explicit…there’s a sense the die was cast very early on” (P4) and from another “…the first time I saw what was in the thing was the day it came out”. (P13)

### The programme stream

The programme stream is focused on implementation [35]. This section will examine oral health policy implementation successes and failures in Ireland during the study period in addition to considerations for the implementation of ‘Smile agus Sláinte’.

Based on both sources of data in this study it can be concluded that the implementation of elements of the ‘Dental Health Action Plan’ and community water fluoridation were successful. As noted in interviews, political support for the DHAP combined with strong dental leadership, collaboration and communication supported implementation. While analysis of documents pertaining to community water fluoridation also illustrate political support, investment in research and proactive engagement by way of the Forum on Fluoridation in response to public concerns [51].

However, a persistent finding emerging from the documentary analysis was the failure to implement a variety of recommendations, guidelines and a national oral health policy during the study period [41, 42, 52, 62–64]. This was especially evident with respect to the outcomes of national oral health surveys [43, 44, 73], clinical guidelines [41, 62–64], recommendations to expand dental specialist lists [42] and the failure to publish the draft national oral health policy in 2009 [52].

This was described by an interviewee: “The failure to invest, particularly…from 2000 to 2010…is really important, it was possible…a number of initiatives were there but getting them over the line was next to impossible” (P4) and another: “So as the Irish economy went up, went down, went sideways… There were opportunities to do things” (P8).

The absence of a Chief Dental Officer (CDO) in the Department of Health along with the formation of the HSE in 2005 were suggested by interviewees as contributing factors to non-implementation: “It was a lost decade… from the time the CDO left”(P4) and “…the formation of the HSE. All of that was taking a lot of energy in the Department, dentistry wouldn't have been even on the radar” (P12).

An assessment of documents found that efforts to review the DTSS scheme failed in 2007 when legal concerns raised by the HSE and Department of Health with respect to negotiation with the Irish Dental Association triggered the Associations withdrawal from discussions [74]. Analysis also points to issues with respect to probity assurance within the DTSS scheme [75] and resistance from the dental profession during negotiations. An interviewee familiar with events stated: “It was just so slow…they were…trying to block anything…”(P12) and another: “Between Department of Health, HSE and Dental Association. No party emerges from it with credit…it was a missed opportunity”. (P4).

‘Smile agus Sláinte’ commits to developing an implementation plan as a “priority” [21]. The time frame for implementation is from 2019–2026 with agreement on an implementation plan with lead partners described as the “initial focus” [21]. In 2021, this remains unpublished with the DoH stating the emergence of the COVID-19 pandemic had caused the policy roll out to be “delayed” [39].

The impact of the pandemic was also of concern for interviewees: “I'd be sceptical about anything happening… when this is all over…that’s gona [going to] look like nothing compared to mental health, cancer, missed appointments…” (P13).

While relationships between dental practitioners and the DoH were again challenged during the pandemic relating to the provision of PPE: “They were promised PPE, and they're treating medical card patients, they were solidly promised PPE. And that promise was not delivered…where's the trust going to be now for the oral health policy?” (P9).

A consistent finding from interviews was the need for better engagement between all stakeholders for implementation to progress: “…the way forward is you start by talking but unfortunately talking just won’t do it…they have to change the mood…buy goodwill… early wins…what’s not contentious” (P4). The absence of public dental funding was also highlighted: “The State is going to have to support dentistry in an unprecedented way…”(P1) and “…the biggest obstacle they are going to face is, is one of the biggest obstacles they always faced…it requires significant additional resources”. (P10).

The importance of political support for policy implementation and support for senior officials deemed responsible for it was raised by one interviewee: “The Chief Dental Officer needs support at the highest level in the Department of Health, including from the Minister, who must want the plan to succeed and state it publicly and not just lip service” (P11). While the need for urgency was strongly advocated for: “We're at a breaking point, both in terms of the children's and adults’ services, now's the time to start the policy…if we mess this up, we lose one of the best opportunities we’ve ever had” (P1).

## Discussion

The low political interest in oral health and the neglect of oral health policy as illustrated by this analysis of the Irish experience reinforces international literature in this area [2, 4]. Political power is one of the most important factors in determining the priority given to oral health policy [76, 77]. While the failure to incorporate oral health onto political agendas may exacerbate oral health inequalities, a key feature of the ‘problem stream’ in Ireland [5].

Oral health has not featured prominently on the Irish political radar for the vast majority of the 27 years under scrutiny. Legislation governing the dental profession stems from 1985 [78] and the failure to update national oral health policy for over twenty five years, both strongly reinforce this international finding [21]. The ‘Dental Health Action Plan’ published in 1994 was initially successful [32]. Its publication coincided with the tenure of a left-leaning health minister who, from analysis in this research, is given credit for providing political backing for policy implementation. This is reflective of more recent international literature showing that where left-wing parties are in power there is greater support and probability of delivering universal health coverage and more progressive health policies [79]. Furthermore, the policy problem of poorer oral health among lower socioeconomic groups [68], along with demands for orthodontic care achieved prominence at the same time a national health policy was in development. In other words, there was an “open window of opportunity” for policy change [80]. Policy agenda setting theory proposes that “multiple streams” must align for issues previously not high on the political radar to become visible [80]. This was evident in 1994 and further supported by alignment between the Department of Health and the dental profession [32].

However, there was a failure to build on the ‘Dental Health Action Plan’ and political attention diminished during the following decades despite the opportunity for action. When the Irish economy experienced exponential growth during the ‘Celtic Tiger’ years (2000–2007), the health system benefited from increased spending [81]. There was increased investment in dental research [43, 44, 73], in commissioned reports evaluating dental services [42, 45, 47, 75] and a Draft National Oral Health Policy [52]. However, there was a failure to increase coverage for dental care [12], to fulfil most of the conclusions and policy recommendations made [41, 45, 62–64, 75] or to incorporate oral health into general health policies [20, 58]. This mirrors findings internationally where a failure to fully implement stated commitments to oral health coupled with the fragmented integration of oral health into broader health systems is indicative the low importance attributed to it by decision makers [25].

This lack of political priority is not unique to Ireland with global oral health suffering from limited political attention [2, 6]. Oral diseases disproportionately impact marginalised groups, have low mortality, and are often considered inevitable which can influence their political importance [6, 76, 77]. This persistent underestimation from a political perspective of the burden and impact of oral diseases and an unawareness of their harm creates a situation where high level acknowledgements of oral health problems by political leadership are “toothless” [77]. The exception in the Irish context is the political interest in orthodontic care. Evidence suggests that demand for orthodontic treatment is rising as the health and expectations of populations improve, along with increased concerns regarding dental aesthetics [82]. In Ireland, access to publicly funded orthodontic care is determined by treatment need rather than income level, with research showing that children of lower socioeconomic status are less likely to have had orthodontic treatment than their better off counterparts [73]. When considering the prominence afforded to orthodontics, a DoH commissioned report pointed to the pressures placed on politicians by “middle class parents” seeking orthodontic care for children as a possible reason for the focus [42]. In 2021 during a governmental committee meeting on the difficulties experienced by low-income adults accessing dental care, participating politicians discussed orthodontic waiting lists for children, with a dental professional representative cautioning the lack of access to basic oral healthcare as a “much bigger impediment to child health”[66].

The dominance of orthodontics across the political discourse in the absence of ‘essential’ primary oral health care services is particularly stark and poses the question, what constitutes essential oral healthcare? The categorisation of ‘essential oral healthcare’ is a topical issue in the global domain particularly in the aftermath of the COVID-19 pandemic [83]. Defining essential oral health care is considered a “formidable and highly pertinent challenge” [83]. However, the engagement of all stakeholders in determining this definition is strongly advocated for, as is ensuring services are rooted in the “immense unmet oral health needs” across populations with “political decisions grounded in science and evidence” [83].

The importance of coalescence and a joint understanding between political stakeholders and oral health advocates is echoed throughout the literature [76, 77]. The role of those actors in oral health policymaking is crucial [24, 26, 76, 84] with respect to their use of evidence, in setting the agenda, emphasizing the importance of oral health, and mobilizing resources [85].

An important finding of this study is the lack of policy champions across the oral health policy landscape in Ireland at critical intervals (Table 4). The absence of a Chief Dental Officer in the Department of Health during key periods and an oral health advocate in a position of national authority in the HSE resulted in a lack visibility for oral health in decision making processes [45]. This is highlighted in the DoH commissioned evaluation of the public dental service (PDS): “The leadership deficit in both the Department of Health and the HSE is an impediment to developing and delivering the service” [47]. A consequence of insufficient leadership and a lack of oral health specialist expertise within policymaking bodies seen internationally is the failure to publish national oral health policies [77]. This was also evident in the Irish context with the failure to publish the ‘Draft National Oral Health Policy’ in 2009 and recognise critical inequalities pertaining to the public dental system which still dominate in 2021.

Research examining the power of actors in the oral health policy context also finds the private sector strongly influences the policy making debate [76]. In the absence of skilled dental public health specialists, advocacy initiatives can fall to professional representative groups with a subsequent focus on curative interventions rather than prevention [77]. This is found in Ireland, where dental public health is not a recognised specialty and the numbers of specialists are low [42]. The Irish Dental Association (IDA) represents the interests of private dental practitioners and salaried dentists working in the public system. The collapse of contract negotiations between the IDA, HSE and Department of Health in 2007 was considered a failure across all parties, however, the resistance of the profession was highlighted. This pursuit by the profession of their independent agenda is reflected in international research where efforts to prioritise oral health can be dominated by the private sector perspective [76].

However, the influence of the dental profession has been significantly restricted since the introduction of austerity measures in 2010. A key component of oral health policymaking is the facilitation of conversations and dialogue between the different participants in the policy process [25]. This research finds that insufficient engagement has taken place with all dental professionals during the development of ‘Smile agus Sláinte’. Stakeholder engagement is “critical” in delivering healthcare change and supporting implementation [86]. International evidence highlights that efforts to transform health systems are more successful when healthcare professionals are engaged, leading to improved clinical outcomes, patient safety, care quality and financial performance [87].

To progress policy implementation and reform in Ireland, the agendas of the DoH and dental professionals require alignment and leadership to unite the policy community. International evidence emphasises the importance of developing a consensus among stakeholders on a shared vision and appropriate strategies [76]. While cautioning that “simple document-based policy reforms” may not have the desired impact without widespread stakeholder support to carry it through to full funding and implementation [25].

The emergence of the COVID-19 pandemic and likely future impacts on health spending has generated concerns amongst participants in this study regarding potential implementation. The pandemic also highlighted the position of oral health within the broader health system. Ireland, like many countries, limited dental services to “emergency practice” in March 2020 [83, 88]. By May 2020 “routine dentistry” recommenced under new legal regulations with dental services classified as an “essential service for Irish society” [89]. Historically oral health has often been assigned to individual responsibility [90]. However moving forward, it must be an integral component of any health system. The emergence of “essential oral healthcare” as a consequence of the pandemic must be defined and further harnessed in supporting future health system reform, particularly in the realm of UHC [83].

This research finds that oral health policy in Ireland is “path dependant” illustrated by its association to previous decisions and existing institutions [91]. The Irish path has been one of providing minimal and emergency cover for some population groups, without universal coverage and a failure to focus on prevention and early intervention [12, 21]. To avoid repeating historical mistakes, evidence suggests that “strong conjunctural forces”are necessary to move policy away from its existing path onto a new trajectory [91]. While ”windows of exceptional opportunity” that determine the ways a political system reacts to policy are required [91]. In May 2021, The World Health Assembly adopted a historic resolution on oral health [92]. The central recommendation echoes calls internationally for the inclusion of oral health under the universal healthcare agenda [2, 10, 90]. A the time of writing in July 2021, Irish political attention appears focused on the adult medical card scheme (DTSS) (Table 3) where the numbers of dentists participating in the publicly funded scheme are rapidly declining and patients are struggling to access dental care [39]. In April 2021, the DoH stated: “the pandemic has caused…the roll-out of the policy to be delayed…and the proposed contract review (of the DTSS scheme) deferred” [39].

The impetus for inclusion of oral health within UHC emerging worldwide, for which there is an agreed political consensus in Ireland [20], could provide a platform for oral health policy in Ireland and the opening of a window of opportunity to bring about major oral health system reform.

### Limitations/strengths/potential contributions

The key strengths of this paper include the use of a structural framework following best practice recommendations for policy analysis [23, 35] and the use of multiple sources of data [30]. Interviews are considered useful in policy analysis to provide rich information, particularly of a sensitive nature. However, interview data can be ambiguous and subjective [93]. To overcome this potential limitation, interview data was continually triangulated with a detailed documentary analysis. Although the sample size for this study was relatively small (n = 13), with greater participation among policymakers sought, owing to the narrow oral health policy landscape in Ireland and the information power [94] generated from the interviews, we are confident of the strength of the findings. Anonymity was granted to each interviewee with all transcripts anonymised and securely stored while the participants identities were known only to the research team. Furthermore, no identifiable quotations will be presented in this or any other publication arising from this analysis. This research presents an original contribution to knowledge, an in-depth analysis of oral health policy in Ireland, the first study of its kind. This research highlighted the limited number of studies analysing oral health systems and policies along with the political and external influences which shape them. It is incumbent on the authors of this paper to urge for greater research in this area and echo organisations such as the International Association for Dental Research in advocating for more implementation and oral health systems research [95].

## Conclusion

This research clearly finds that oral health is not a political or policy priority in Ireland. The publication of ‘Smile agus Sláinte’ in 2019 provides an opportunity for much needed reform of the public dental system. However, to avoid repeating historical mistakes successful reform will require greater political interest than experienced to date, strong political will, and a major focus on implementation, including positive engagement with oral health professionals. Internationally, calls have been made for the inclusion of oral health as part of the universal healthcare agenda. Ireland’s national health policy ‘Sláintecare’ has agreed political consensus and an implementation plan in progress to deliver universal healthcare. In September 2021, the two most senior personnel responsible for Sláintecare resigned. In the aftermath of this, the Irish Government recommitted its promise of universal healthcare, however the extent that it will be implemented remains unclear. Incorporating oral health reform as an integral part of any future universal healthcare implementation may provide the opening of a window of opportunity. Learning from this research, genuine engagement with all stakeholders in the development of a detailed implementation strategy for oral health  system reform under the remit of universal healthcare is urgently required before the window closes yet again.

## Data Availability

The datasets generated and analysed during the current study are not publicly available to maintain the anonymity guaranteed to study participants but may be available from the corresponding author on reasonable request.

## Abbreviations

UHC: Universal Healthcare
DoH: Department of Health
HSE: Health Service Executive
OHP: Oral Health Policy
CWF: Community Water Fluoridation
PDS: Public Dental Service
DTSS: Dental Treatment Services Scheme
DTBS: Dental Treatment Benefit Scheme

## Footnotes

1 _P refers to ‘Participant’ with each interview participant assigned a code from P1-P13._

2 _In Ireland children attend their first-level education in primary schools (also called national schools) between the ages of 4 or 5 years and 12 years of age. Each year of education is referred to as a primary school class. The public dental services target children for assessment depending on which school class they attend._
